# Establishment of an HPLC Fingerprint and Cluster Analysis for Miao Ethnic Medicine *Osbeckia opipara*

**DOI:** 10.5812/ijpr-146396

**Published:** 2024-08-04

**Authors:** Qiang Su, Ting Su, Yun Lu, Min Wu, Song Huang, Shouneng Chen, Jiang Liang, Zhenxiang An

**Affiliations:** 1 Guizhou University of Traditional Chinese Medicine, Guiyang, Guizhou Province, China; 2 Fenggang County Traditional Chinese Medicine Hospital, Zunyi, Guizhou Province, China; 3 Changsha Jingyi Pharmaceutical Technology Co., Ltd, Changsha, Hunan Province, China; 4 Department of Rheumatology and Hematology, The First Affiliated Hospital of Guizhou University of Traditional Chinese Medicine, Guiyang, Guizhou Province, China; 5 Department of Gastroenterology, The First Affiliated Hospital of Guizhou University of Traditional Chinese Medicine, Guiyang, Guizhou Province, China

**Keywords:** Miao Ethnic Medicine, *Osbeckia opipara*, High-Performance Liquid Chromatography (HPLC), Fingerprint Profile, Zhengshi Wu

## Abstract

*Osbeckia opipara*, a traditional Miao medicine, is commonly used by the renowned national-level Chinese Traditional Medicine practitioner Zhengshi Wu for the treatment of diarrhea due to its strong antioxidative, anti-inflammatory, and antidiarrheal effects. This study aimed to establish a high-performance liquid chromatography (HPLC) fingerprint for *O. opipara* to provide new evidence and technical means for the scientific evaluation and effective quality control of *O. opipara*. The procedure involved isolation with a Nano ChromCore C18 column (250 mm × 4.6 mm, 5 μm), using a gradient elution of 0.1% formic acid in water and 0.1% formic acid in acetonitrile as the mobile phase, with a flow rate of 1.0 mL/min, a column temperature of 30°C, an injection volume of 10 μL, and detection at a wavelength of 254 nm. Under these chromatographic conditions, fingerprint analysis was conducted on 11 batches of *O. opipara* collected from different origins. The National Pharmacopoeia Committee developed the 'Chromatographic Fingerprint Similarity Evaluation System' (2004A version) for automated comparison, similarity computation, and analysis of chromatographic data. The results revealed 13 common peaks across the 11 batches of *O. opipara* samples, with a similarity to the automatically generated reference spectrum exceeding 0.9. SPSS 26.0 software was used to conduct cluster analysis on the peak areas of the 13 common peaks. The observations indicated that the reference spectrum generated from the 11 batches could serve as the standard fingerprint profile for *O. opipara*, providing sufficient characteristic information extraction.

## 1. Background

Born in 1940 in Kunshan, Jiangsu, China, Zhengshi Wu initiated his training under his father, Yuwen Wu. Notably, he is the nineteenth-generation successor of the 'Chetang Wu Family's School of Wind Disorders'. He graduated from Shanghai Jiao Tong University School of Medicine and completed his postgraduate studies at Guizhou University of Traditional Chinese Medicine (1). As a chief physician and expert in traditional Chinese medicine, he mentors the second cohort of veteran Traditional Chinese Medicine (TCM) experts, is the recipient of a State Council special allowance, and leads national and provincial initiatives to preserve TCM expertise. His expertise in Miao medicine, specifically *O. opipara*, has led to breakthroughs in the treatment of conditions such as ulcerative colitis and irritable bowel syndrome.

Miao medicine is a traditional medical system practiced in the Miao ethnic areas of China, encompassing a unique system of medical theories and treatment methods. It features distinctive medicinal practices, particularly in the multi-ethnic regions of Southwest China. One widely used Miao medicine is *O. opipara*, which has long been popular among the Miao communities for its effectiveness in alleviating diarrhea and purging heat for detoxification purposes. Consequently, it has been widely applied for the treatment of diarrheal diseases. Zhengshi Wu, utilizing his profound knowledge of TCM and extensive clinical experience, has systematically explored and applied *O. opipara*, achieving favorable outcomes, particularly in the treatment of ulcerative colitis and irritable bowel syndrome.

*Osbeckia opipara* is derived from the roots and fruits of the plant Jinjinxiang (Malvaceae family), also known as 'Guanzicao', 'Lijiguan', 'Yangtianguan', 'Daoshuilian', 'Gaojiaohonggang', and 'Kuoye Jinjinxiang', a commonly used herbal medicine in Miao areas. Characterized by a cool nature, with sour and astringent tastes, it enters the 'heat meridian'. Its primary functions are to clear heat, promote diuresis, detoxify, stop bleeding, and attenuate diarrhea. Thus, it is predominantly used for the treatment of damp-heat dysentery, diarrhea, lung heat, cough, and hemoptysis (2).

At present, studies on *O. opipara* are scarce. The primary components identified in the root of *O. opipara* are phenols, tannins, and various other substances, which have demonstrated potent antioxidant, anti-inflammatory, and anti-tumorigenic properties (3-6). However, there is still a paucity of reports on the quality assessment of *O. opipara*. Given the diversity and complexity of chemical components in traditional Chinese medicines, it is challenging to comprehensively reflect the quality of materials using a single or a few index components. The fingerprint technique of traditional Chinese medicine is a comprehensive, quantifiable identification method used to evaluate the quality of Chinese medicine, authenticate its origin, distinguish between species, and ensure consistency and stability. Creating a unique fingerprint for traditional Chinese medicine can provide an in-depth representation of the various chemical components present in Chinese medicine and its formulations, ultimately facilitating the assessment of the quality of ingredients (7-9). The combination of cluster analysis with HPLC fingerprint profiles not only streamlines original data processing but also retains most information, addressing numerous challenges encountered in the multivariate quality evaluation of traditional Chinese medicine (10).

To systematically develop and utilize the Miao medicine *O. opipara*, commonly used by the renowned traditional Chinese medicine practitioner Wu Zhengshi, and to ensure its quality, this study collected samples from 11 batches of *O. opipara*. During the creation of HPLC fingerprint profiles, cluster analysis was employed to examine variations among individual batches, identify the chemical components responsible for these differences, and elucidate the variability among different batches of *O. opipara*. 

## 2. Objectives

Overall, this study is anticipated to provide a theoretical reference for quality control and the exploration of the pharmacodynamic material basis in *O. opipara*.

## 3. Methods

### 3.1. Instruments

Instruments used in this study included a versatile high-speed grinder procured from Zhejiang Hongjingtian Industrial & Trade Co., Ltd., a numerical control ultrasonic cleaner model KQ-300DE acquired from Kunshan Ultrasonic Instruments Co., Ltd., a high-performance liquid chromatograph LC-20AT obtained from Shimadzu Corporation in Japan, and a laboratory-specific ultrapure water machine purchased from Sichuan Walter Co., Ltd.

### 3.2. Reagents

Methanol (Tianjin Concord Technology Co., Ltd.); Acetonitrile (Tianjin Concord Technology Co., Ltd.); Formic acid (Tianjin Komio Chemical Reagent Co., Ltd.); Tannic acid (Chengdu Mansite Biotechnology Co., Ltd.); Ultrapure water (self-prepared).

### 3.3. Medicinal Materials

Professor Zhou Jing from the School of Pharmacy at Guizhou University of Traditional Chinese Medicine confirmed the medicinal ingredients of *O. opipara* collected from various regions in Guizhou, including Guiyang, Zunyi, Liupanshui, Bijie, and Anshun. The voucher specimen (20200816) was archived at the Herbarium of Guizhou University of Traditional Chinese Medicine. All samples were processed products of the dried fruits and roots of *O. opipara*. Details are listed in Table 1. After pulverization, the medicinal materials were sieved through a 50-mesh screen, and the resulting powders were stored in a desiccator in a cool, dry environment.

**Table 1.** Information on *Osbeckia opipara*

No.	Origin	Altitude (m）	Processing Method	Remarks
**S1**	Huaxi District, Gaopo Township, Shuitang Village, Guiyang City, Guizhou Province	1340	Drying	Self-collected
**S2**	Longgang Town, Kaiyang County, Guiyang City, Guizhou Province, Doupeng Mountain	1228	Drying	Self-collected
**S3**	Yushe National Forest Park, Shuicheng County, Liupanshui City, Guizhou Province	2266	Drying	Self-collected
**S4**	Huangguoshu Town, Zhenning Buyi and Miao Autonomous County, Anshun City, Guizhou Province	1090	Drying	Self-collected
**S5**	Tunshang Village, Zhenfeng County, Qianxinan Buyi and Miao Autonomous Prefecture, Guizhou Province	910	Drying	Self-collected
**S6**	Xiaoshui Township, Tongzi County, Zunyi City, Guizhou Province	1152	Drying	Self-collected
**S7**	Weixin Town, Nayong County, Bijie City, Guizhou Province	1692	Drying	Self-collected
**S8**	Jiaozi Mountain, Yushetown, Shuicheng County, Liupanshui City, Guizhou Province	2038	Drying	Self-collected
**S9**	Dalongjing Village, Baixing Town, Nayong County, Bijie City, Guizhou Province	1632	Drying	Self-collected
**S10**	Gaopo Miao Ethnic Township, Huaxi District, Guiyang City, Guizhou Province	1459	Drying	Self-collected
**S11**	Changpo, Ziyun Miao and Buyi Autonomous County, Anshun City, Guizhou Province	1264	Drying	Self-collected

## 4. Results

### 4.1. Preparation of Solutions

#### 4.1.1. Preparation of Reference Solution

A pre-defined amount of tannic acid reference substance was weighed and dissolved in methanol to yield a solution containing 1.084 mg of tannic acid per milliliter. This solution was then stored at 2 - 8°C.

#### 4.1.2. Preparation of Test Sample Solution

One gram of *O. opipara* medicinal material powder was weighed and added to 10 mL of methanol, and the mixture was ultrasonicated for 30 minutes. The mixture was then filtered, and the filtrate was transferred into a 10 mL volumetric flask, thoroughly mixed, and filled up to the mark with methanol. The solution was subsequently filtered through a 0.45 μm microporous membrane to collect the filtrate, which served as the test sample solution.

### 4.2. Optimization of Chromatographic Conditions

#### 4.2.1. Investigation of Optimal Flow Rate

**Figure 1.**
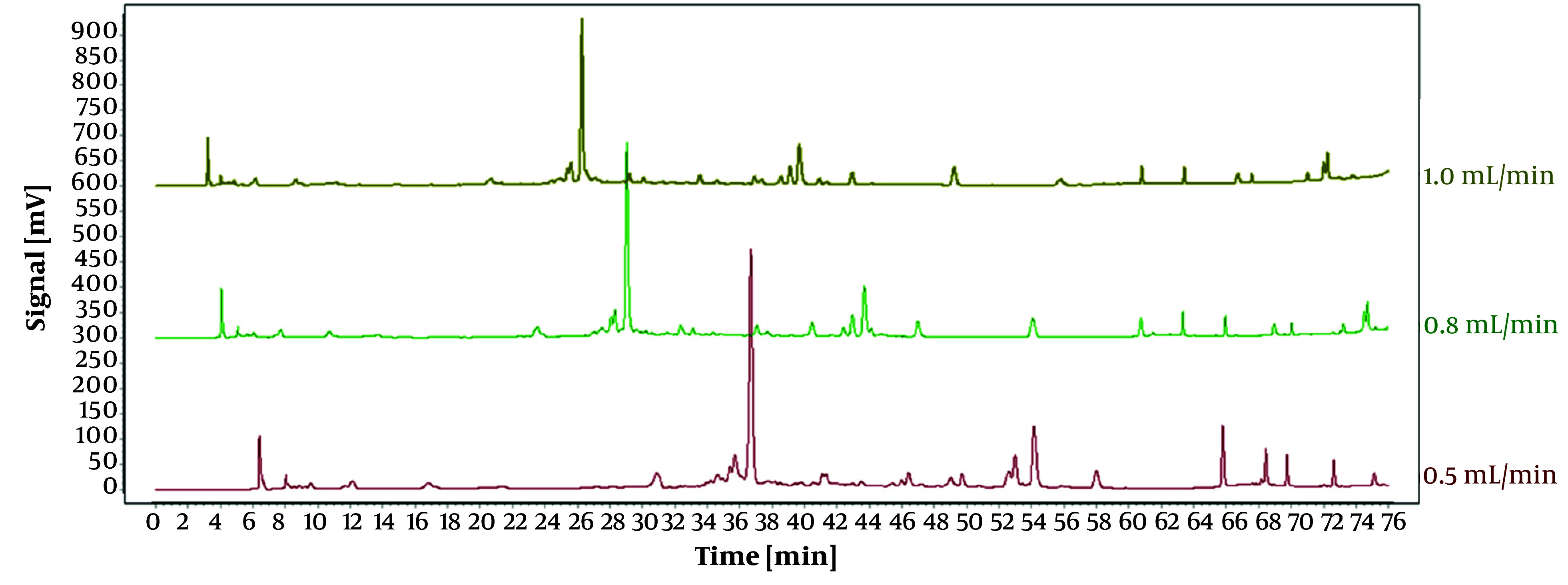
High-performance liquid chromatography (HPLC) chromatograms of *Osbeckia opipara* at different flow rates

As displayed in Figure 1, the impact of three varying flow rates (0.5 mL/min, 0.8 mL/min, and 1.0 mL/min) on the experimental outcomes was investigated under identical conditions. The duration of each peak separation was prolonged at flow rates of 0.5 mL/min and 0.8 mL/min. Conversely, the peaks exhibited enhanced intensity, stability, and separation and eluted more rapidly at a flow rate of 1.0 mL/min. Hence, a flow rate of 1.0 mL/min was selected for the subsequent experiments.

#### 4.2.2. Investigation of Injection Volume

**Figure 2.**
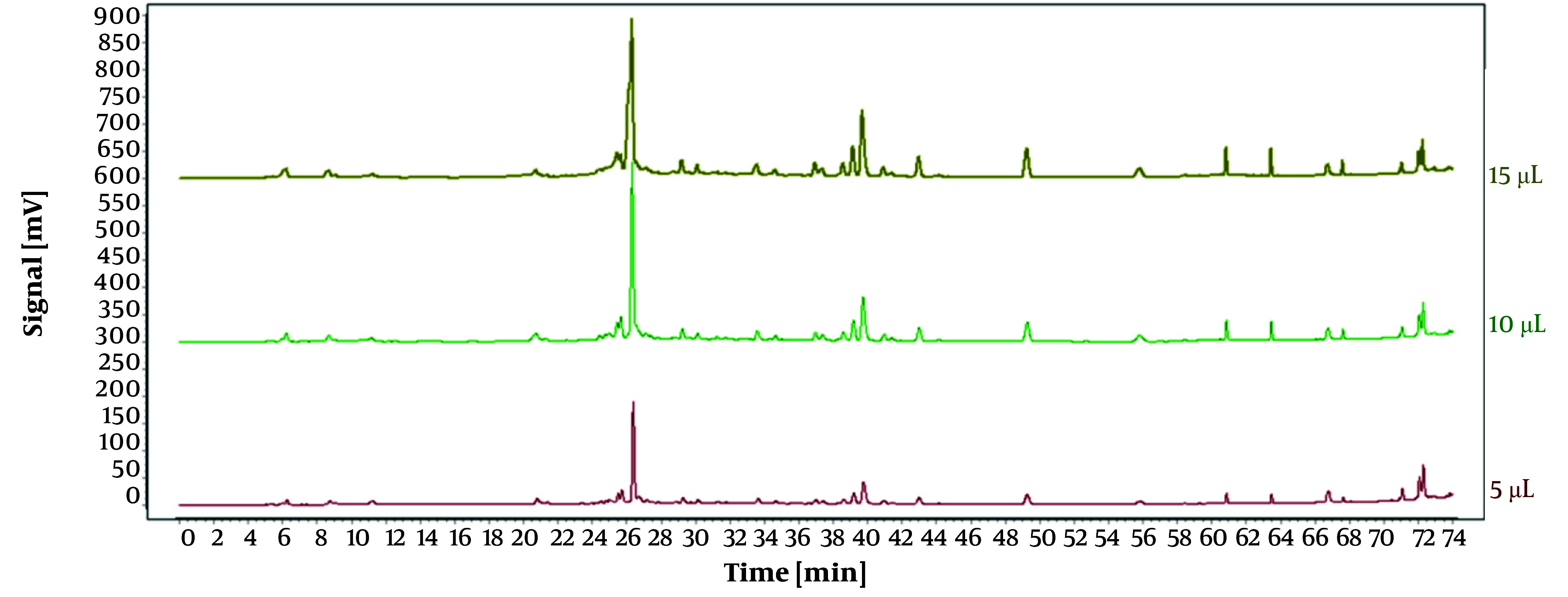
High-performance liquid chromatography (HPLC) chromatograms of *Osbeckia opipara* at different injection volumes

Under identical conditions, the impact of different injection volumes (5 µL, 10 µL, and 15 µL) on the experiment was assessed, with results illustrated in Figure 2. Using an injection volume of 10 µL, the peaks demonstrated enhanced intensity, stability, and separation. Consequently, an optimal injection volume of 10 µL was selected.

#### 4.2.3. Investigation of Mobile Phase

**Figure 3.**
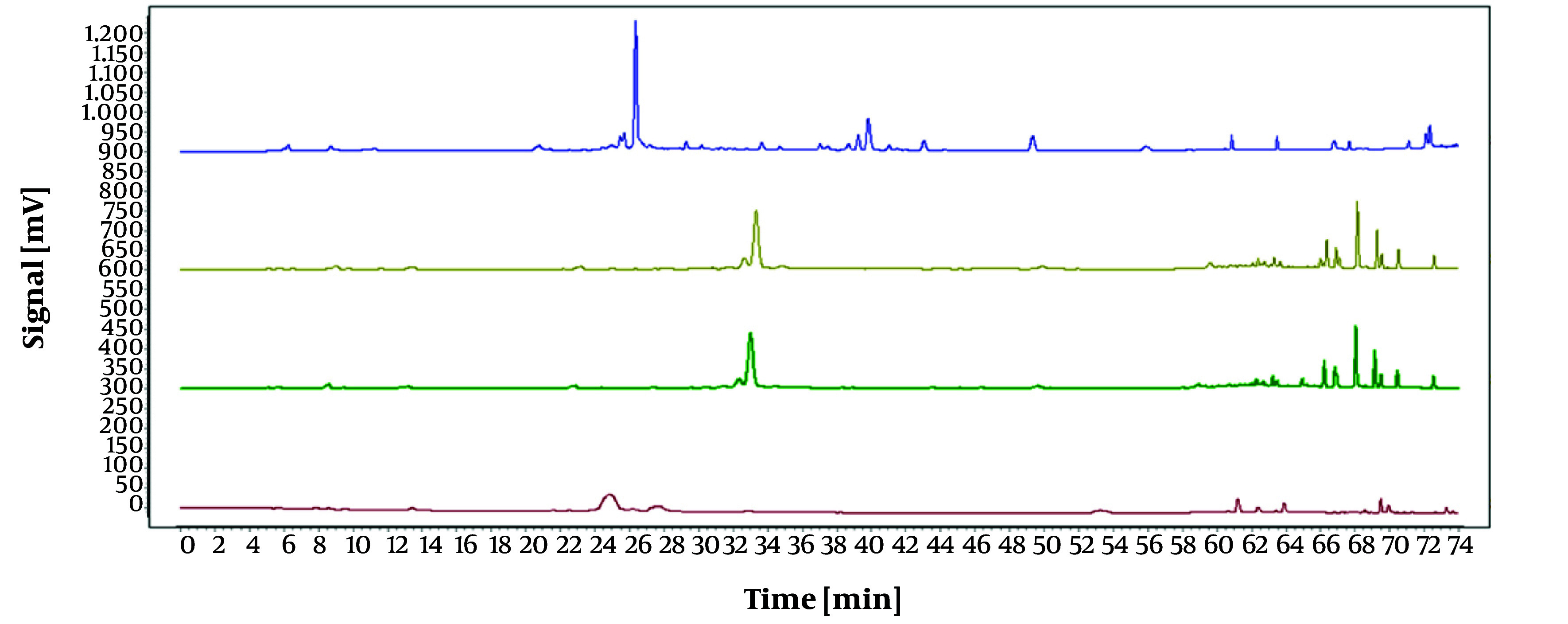
High-performance liquid chromatography (HPLC) chromatograms of *O. opipara* with different mobile phases.

The impacts of four distinct mobile phase setups were examined under identical conditions: Water-methanol (S1), water-methanol with 0.1% acetic acid (S2), water-methanol with 0.1% formic acid (S3), and water-0.1% formic acid in acetonitrile with 0.1% formic acid (S4). The results are depicted in Figure 3. The solution containing 0.1% formic acid in water and 0.1% formic acid in acetonitrile effectively separated all components with improved peak intensity, stability, and resolution. Hence, this setup was chosen as the mobile phase system for the subsequent experiments.

#### 4.2.4. High-performance Liquid Chromatography Chromatographic Conditions

Following multiple optimization experiments, the most effective chromatographic parameters were determined to be as follows: A Nano ChromCore C18 column (250 mm × 4.6 mm, 5 μm), a constant column temperature of 30°C, an injection volume of 10 µL, detection at a wavelength of 254 nm, a flow rate of 1.0 mL/min, and a mobile phase A containing 0.1% formic acid in water and mobile phase B comprising 0.1% formic acid in acetonitrile. The gradient elution conditions are summarized in Table 2. 

**Table 2.** Gradient Elution Program

Time (min)	0.1% Formic Acid in Water (Phase A)	0.1% Formic Acid in Acetonitrile (Phase B)
**0.01**	95%	5%
**5**	95%	5%
**8**	90%	10%
**10**	87%	13%
**45**	82%	18%
**50**	82%	18%
**60**	55%	45%
**75**	10%	90%
**85**	95%	5%

### 4.3. Methodological Validation

#### 4.3.1. Evaluation of Precision

**Figure 4.**
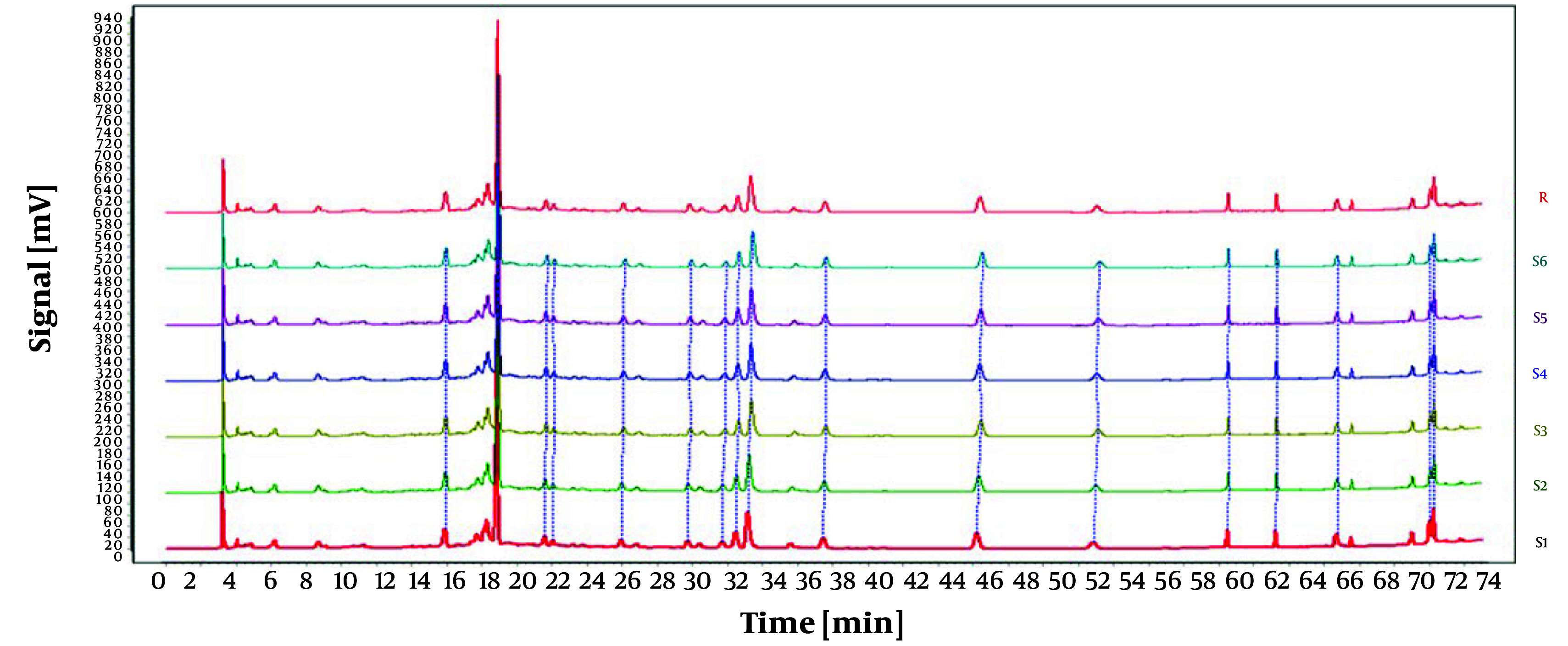
Precision experiment

The test sample solution was prepared as described in section '3.1.2' and injected six times consecutively following the chromatographic conditions outlined in section '3.2.4'. The results are delineated in Figure 4. Interestingly, the Relative Standard Deviation (RSD) for the relative retention times of all shared peaks fell within the range of 0.03% to 0.3%. As presented in Table 3, the correlation coefficients all exceeded 0.99, with an RSD of the correlation coefficient at 0.4%, indicating satisfactory precision. The calculation formula is: 


$$RSD\left(\%\right)=\left(\frac{SD}{\overset{-}{X}}\right)\times 100\%$$

where SD is the standard deviation of the sample and $\overset{-}{X}$ is the average value of the sample. Relative standard deviations (% RSD) were calculated using an Excel sheet (11).

**Table 3.** Results of the Precision Experiment

Injection	Correlation Coefficient	RSD
**1**	0.999	
**2**	0.998	
**3**	0.994	0.4%
**4**	0.999	
**5**	0.999	
**6**	0.999	

#### 4.3.2. Repeatability Test

**Figure 5.**
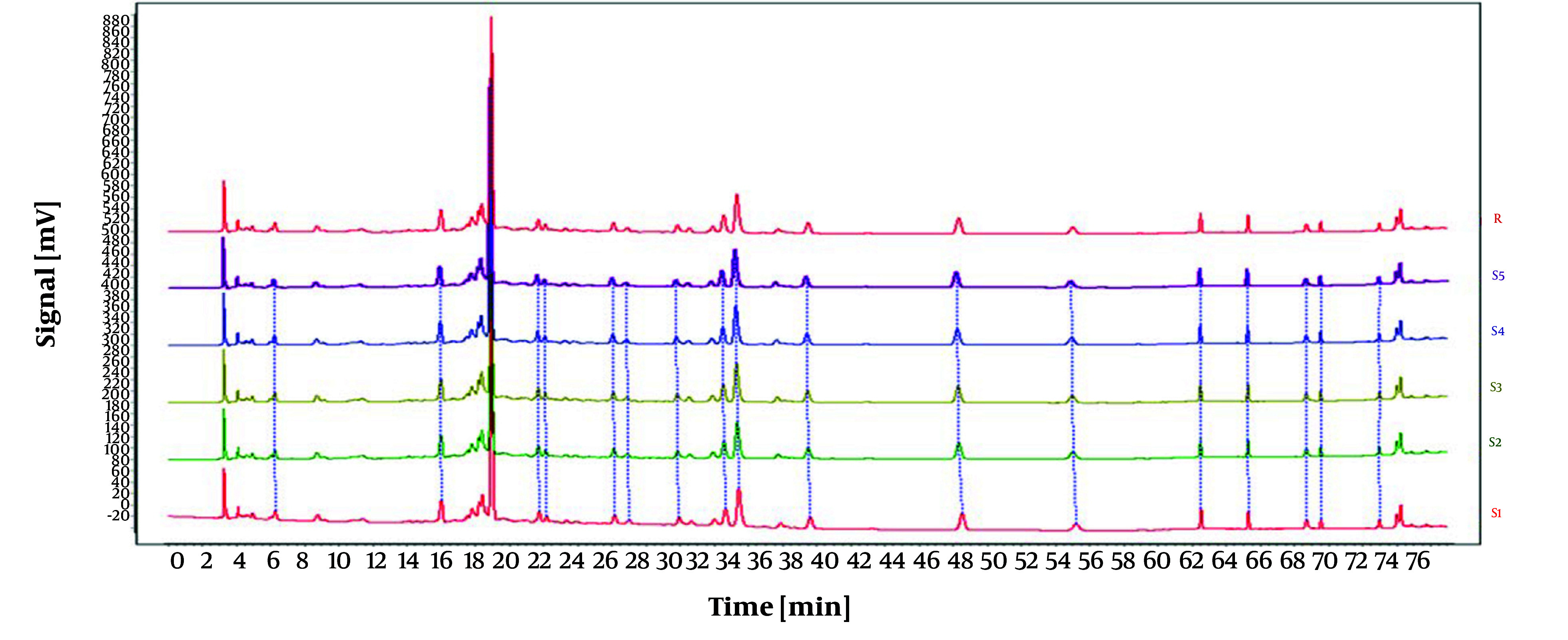
Repeatability experiment

Five parallel test sample solutions were prepared as summarized in section 3.1.2 and subsequently analyzed by injection following the procedures detailed in section 3.2.4. The results are portrayed in Figure 5. Each shared peak exhibited an RSD for its relative retention times below 0.3%. As detailed in Table 4, the correlation coefficients all exceeded 0.98, and the RSD of the correlation coefficients was 1.2%, demonstrating good repeatability.

**Table 4.** Results of the Repeatability Experiment

Sample (Portion)	Correlation Coefficient	RSD
**1**	0.996	
**2**	0.99	
**3**	0.991	1.2%
**4**	0.993	
**5**	0.987	

#### 4.3.3. Stability Test

**Figure 6.**
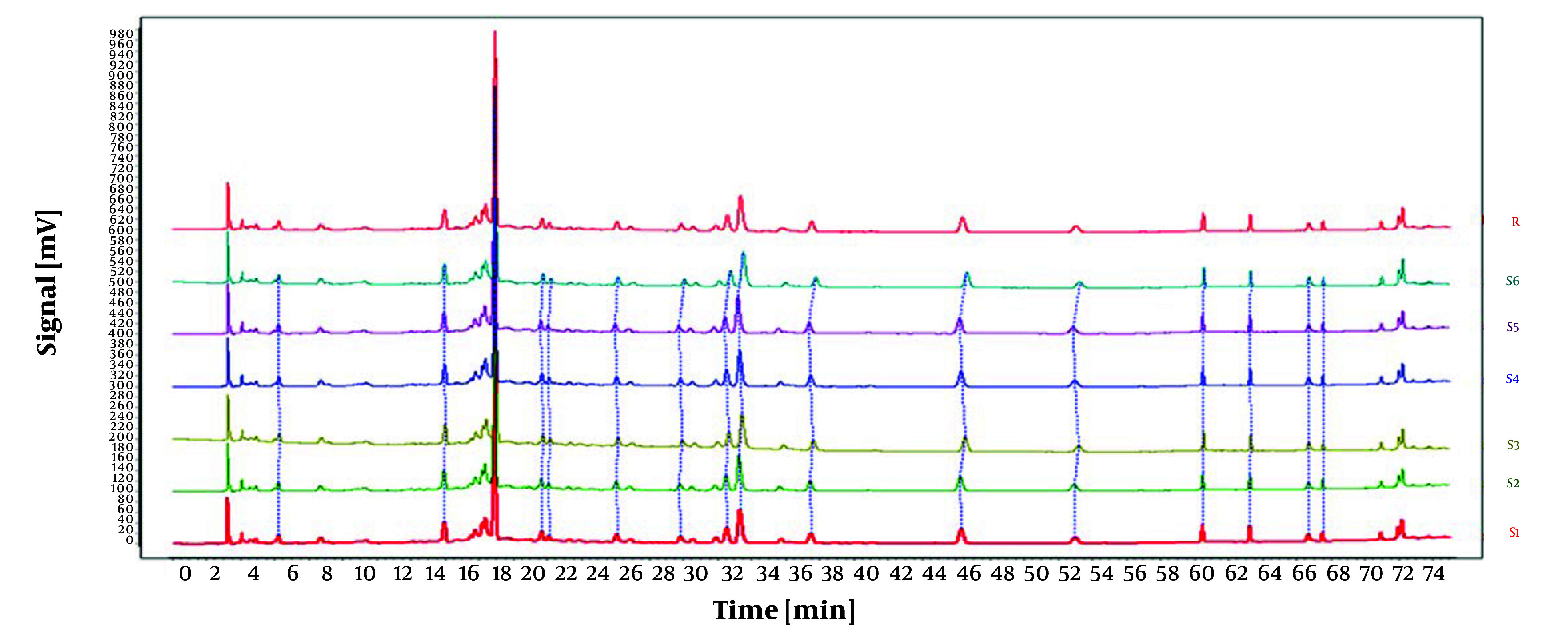
Stability experiment

The test sample solution was prepared as outlined in section 3.1.2 and injected at 0, 2, 4, 8, 12, and 24 hours according to the steps described in section 3.2.4. The results are shown in Figure 6. As anticipated, each shared peak exhibited an RSD for its relative retention times below 0.3%. As presented in Table 5, the correlation coefficients were greater than 0.98, with an RSD of the correlation coefficients at 1.2%, implying good stability.

**Table 5.** Results of the Stability Experiment

Time (h)	Correlation Coefficient	RSD
**0**	0.988	
**2**	0.994	
**4**	0.997	
**8**	0.992	1.2%
**12**	0.992	
**24**	0.986	

### 4.4. Establishment of O. opipara HPLC Chromatographic Fingerprint

**Figure 7.**
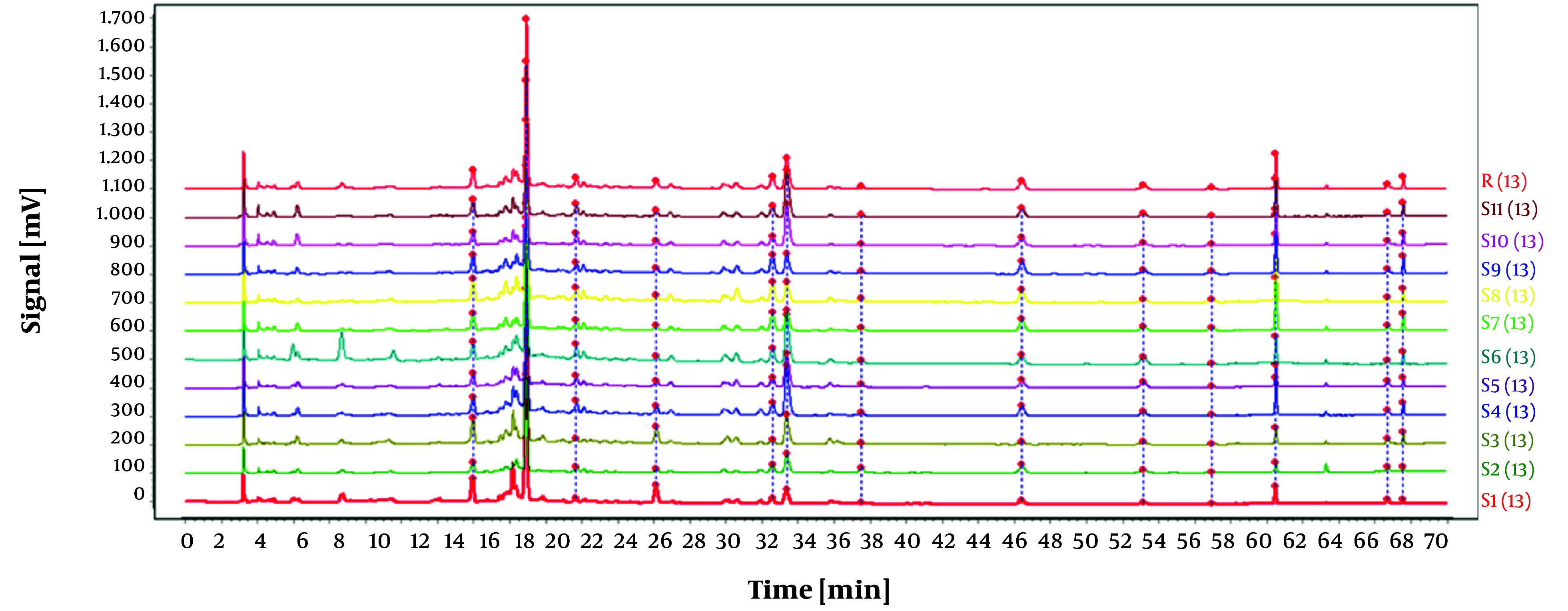
Chromatograms of 11 batches of Guizhou *Osbeckia opipara* medicinal material

In this experiment, samples of *O. opipara* were collected from 11 different regions. The samples were prepared as outlined in section 3.1.2, while the chromatographic conditions were identical to those mentioned in section 3.2.4. The fingerprint profile for the medicinal material was created by importing the chromatograms from the 11 samples of *O. opipara* into the 'Traditional Chinese Medicine Chromatographic Fingerprint Similarity Evaluation System, Version A'. This profile included 13 common peaks (as shown in Figure 7). 

**Figure 8.**
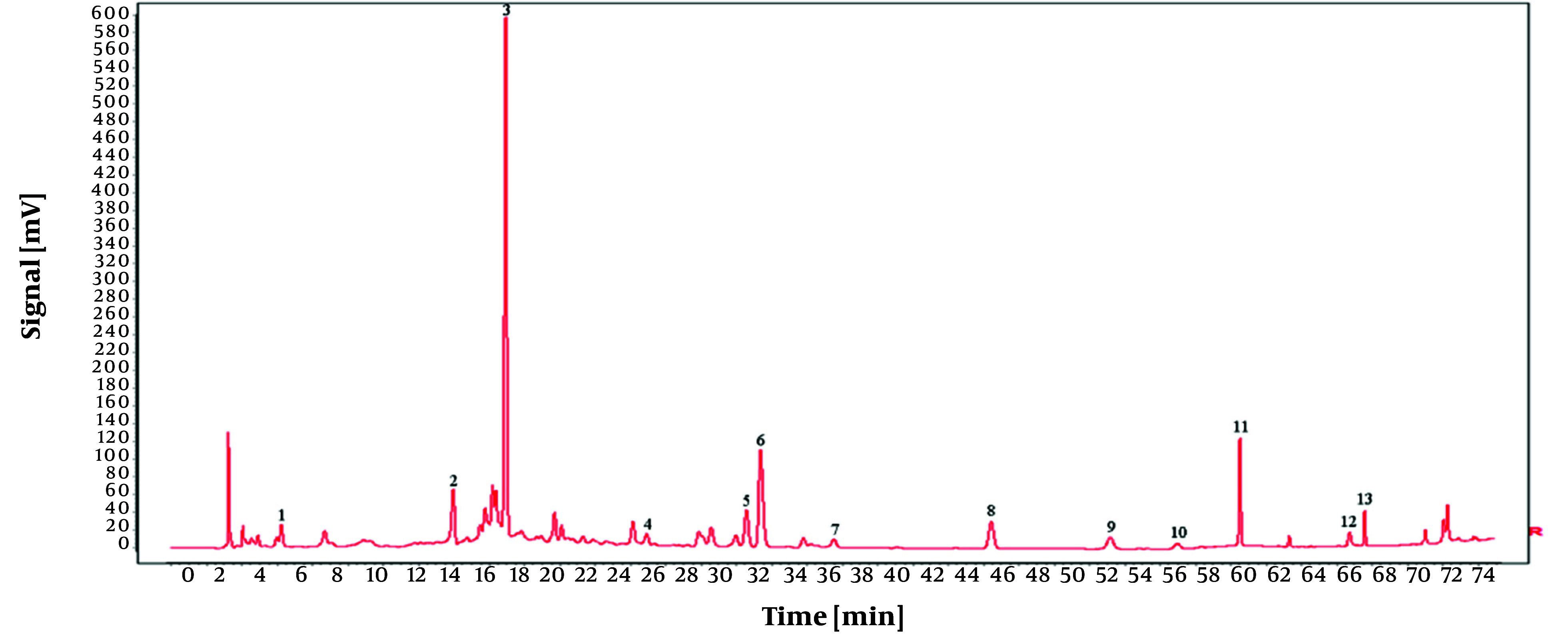
Common pattern diagram of 11 batches of Guizhou *Osbeckia opipara* medicinal material

To establish the characteristic and comprehensive fingerprint, 11 batches of the *O. opipara* samples from different origins were analyzed by HPLC and standardized by the Similarity Evaluation System for Chromatographic Fingerprint of Traditional Chinese Medicine (Version 2004A). There are 13 common peaks in the HPLC fingerprints with good resolution, which were regarded as the reference chromatogram (Figure 8). Among these 13 common peaks, peak 8 was chosen as the reference peak (S) due to its high content, high intensity, and moderate retention time in the *O. opipara* chromatograms. The relative retention time (RRT) and relative peak area (RPA) with respect to the reference peak were then measured (12). The RRT and RPA statistics in HPLC fingerprints were useful for the quality assessment of *O. opipara*. 

**Figure 9.**
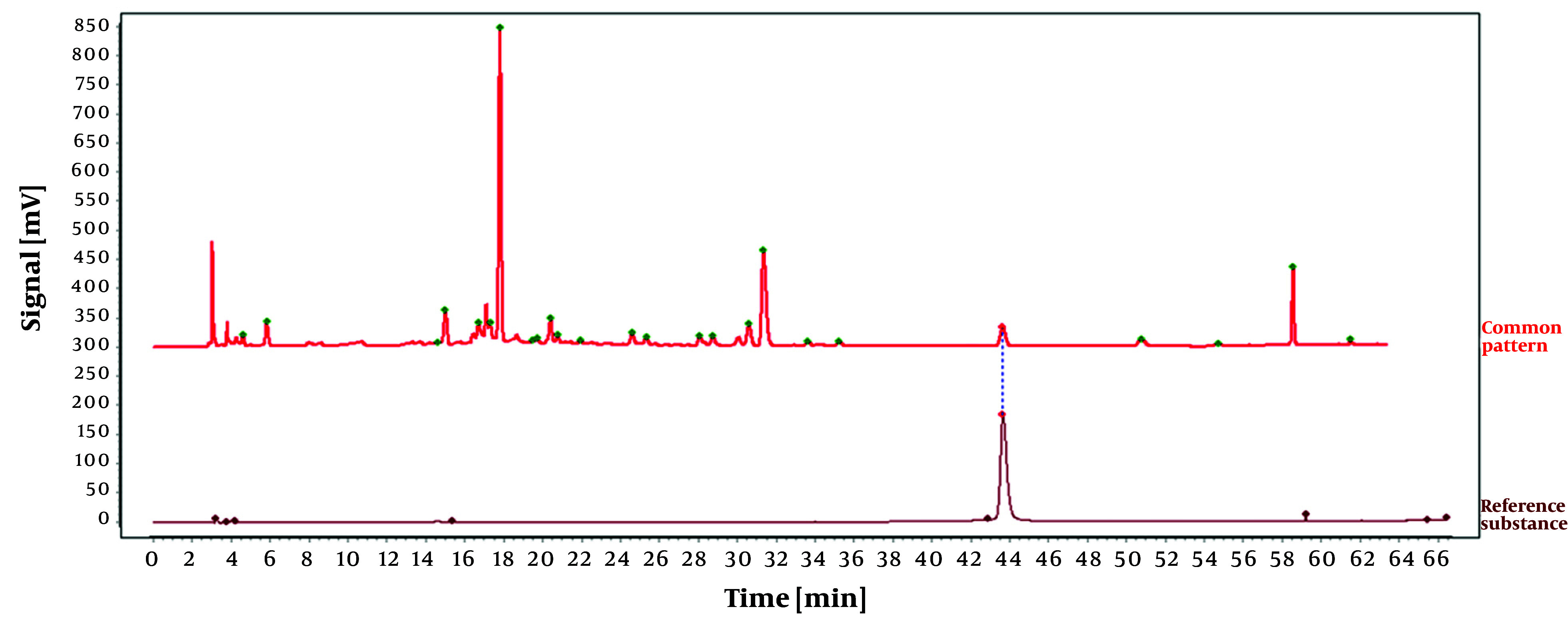
Comparison diagram between reference substance and common pattern diagram

The reference substance was prepared as outlined in section 3.1.1, and the chromatographic conditions were identical to those outlined in section 3.2.4. Additionally, peaks corresponding to the retention time of the reference substance tannic acid were identified by comparing the reference chromatogram with the standard pattern reference chromatogram, as illustrated in Figure 9. The findings demonstrated that the RSD for the relative retention times of all shared peaks was below 0.3%, suggesting that their compositions were nearly identical. However, variations in the peak areas of some peaks suggested that the abundance of components may differ among different batches of *O. opipara*.

### 4.5. Chemometric Methods Analysis of O. opipara Medicinal Material Fingerprint

#### 4.5.1. Similarity Analysis of 11 Batches of O. opipara Medicinal Material Collected from Different Areas

Next, the chromatograms of the 11 batches of *O. opipara* test samples were matched with the reference fingerprint chromatogram. Similarity evaluation was conducted by comparing the peak areas through correlation coefficient similarity, a method derived from the Chinese Pharmacopoeia (13).

As shown in Table 6, the similarity of the 11 batches of *O. opipara* medicinal material all exceeded 0.9, indicating a high degree of consistency.

**Table 6.** Similarity Calculation Results of 11 Batches of *O. opipara* from Guizhou

	S1	S2	S3	S4	S5	S6	S7	S8	S9	S10	S11	Reference Fingerprint
**S1**	1	0.908	0.948	0.886	0.903	0.841	0.911	0.922	0.916	0.846	0.866	0.916
**S2**	0.908	1	0.962	0.978	0.978	0.951	0.966	0.956	0.953	0.959	0.968	0.982
**S3**	0.948	0.962	1	0.958	0.953	0.903	0.958	0.965	0.959	0.925	0.944	0.977
**S4**	0.886	0.978	0.958	1	0.991	0.96	0.958	0.946	0.948	0.991	0.996	0.987
**S5**	0.903	0.978	0.953	0.991	1	0.954	0.957	0.943	0.945	0.982	0.987	0.984
**S6**	0.841	0.951	0.903	0.96	0.954	1	0.909	0.89	0.898	0.957	0.961	0.942
**S7**	0.911	0.966	0.958	0.958	0.957	0.909	1	0.993	0.992	0.945	0.952	0.988
**S8**	0.922	0.956	0.965	0.946	0.943	0.89	0.993	1	0.995	0.922	0.936	0.982
**S9**	0.916	0.953	0.959	0.948	0.945	0.898	0.992	0.995	1	0.926	0.94	0.982
**S10**	0.846	0.959	0.925	0.991	0.982	0.957	0.945	0.922	0.926	1	0.996	0.973
**S11**	0.866	0.968	0.944	0.996	0.987	0.961	0.952	0.936	0.94	0.996	1	0.982
**Reference Fingerprint**	0.916	0.982	0.977	0.987	0.984	0.942	0.988	0.982	0.982	0.973	0.982	1

#### 4.5.2. Cluster Analysis Using SPSS

**Figure 10.**
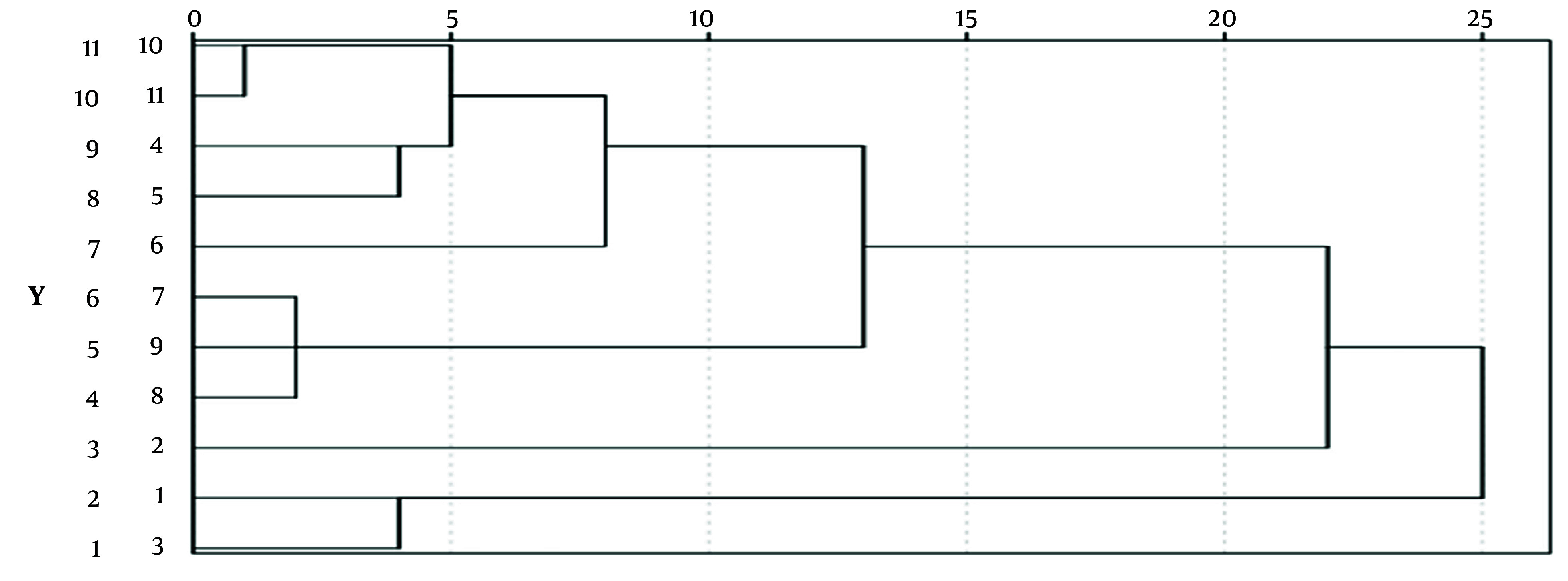
Cluster diagram of 11 batches of *Osbeckia opipara*

Using the peak areas of the common peaks from 11 samples of *O. opipara* as variables, a systematic cluster analysis was conducted using SPSS 26.0 analysis software (Figure 10). The between-groups linkage method was employed, with Euclidean distance as the measure for clustering. At a distance of 25, all objects formed a single large category without further division, indicating marginal differences between *O. opipara* samples originating from different areas (Numbers 1 - 13 in the analysis correspond to the order of *O. opipara* in Table 1). 

## 5. Discussion

In addition to investigating chromatographic conditions, this study also explored the impact of extraction solvents on extraction efficiency. Specifically, the study examined water, methanol, and a water-methanol mixture (20: 80) as extraction solvents. When water was used as the solvent, fewer peaks were separated, and many could not be effectively isolated. The addition of methanol, particularly at an 80: 20 methanol-water ratio, significantly optimized extraction efficiency, allowing for the extraction of several substances. Using pure methanol as the solvent resulted in all components being baseline separated, with peaks exhibiting better intensity, stability, and separation.

This study has laid a preliminary foundation for elucidating the chemical composition and pharmacological characteristics of a specific traditional Chinese medicine. To better understand its chemical makeup and mechanisms, it is essential to utilize sophisticated analytical methods such as Gas Chromatography-Mass Spectrometry (GC-MS) and Liquid Chromatography-Mass Spectrometry (LC-MS). These methodologies are not only capable of identifying a broader spectrum of secondary metabolites but are also instrumental in elucidating their influence on the efficacy of the product.

Furthermore, it is critical to explore the pharmacological actions and mechanisms of action of the products and associated metabolites. While the traditional applications of Chinese medicine offer valuable insights into their potential effects, scientific validation is key. Cellular and animal experiments are crucial to reveal the potential pharmacological actions and underlying mechanisms, which is pivotal for evaluating the clinical applicability of these herbal medicines (14). Additionally, safety assessment is a critical component of research in traditional Chinese medicine. Comprehensive toxicological evaluations, including acute and chronic toxicity studies, are vital to ensuring the safety of the herbal constituents (15). 

Although our research provides a basis for understanding the chemical and pharmacological properties of the medicine, the lack of clinical studies limits our understanding of its effects on the human body. Further clinical trials are warranted to evaluate the effectiveness and safety of *O. opipara* in human subjects.

In summary, our study laid a preliminary theoretical foundation and outlined a research direction for the further exploration of traditional Chinese medicine. Future studies should aim to comprehensively analyze the components, investigate their pharmacological mechanisms and safety profiles, and establish stringent quality control standards in order to provide scientific validation and evidence for the Miao ethnic medicine *O. opipara*, commonly utilized by the esteemed traditional Chinese medicine practitioner Wu Zhengshi for the treatment of intestinal diseases.

### 5.1. Conclusions

This study established the fingerprint profile of *O. opipara* using HPLC. Specifically, a common model was generated using thirteen characteristic peaks that exhibited high responses and favorable separation. The quality of the medicinal material was assessed using the software 'Traditional Chinese Medicine Chromatographic Fingerprint Similarity Evaluation System' developed by the National Pharmacopoeia Committee. The findings indicated that the similarity among the 11 sets of *O. opipara* herbal material exceeded 0.9, suggesting that the reference spectrum derived from these 11 sets could serve as the official fingerprint pattern for *O. opipara*. The approach used in this research provides a theoretical framework for ensuring the quality of *O. opipara*, enabling its assessment and authentication.

## Data Availability

Data will be made available upon reasonable request. The data presented in this study have been uploaded during submission as a supplementary file and are openly available for readers upon request.
